# Acute exacerbations of COPD versus IPF in patients with combined pulmonary fibrosis and emphysema

**DOI:** 10.1186/s12931-020-01432-x

**Published:** 2020-06-30

**Authors:** Massa Zantah, Yaniv Dotan, Chandra Dass, Hauqing Zhao, Nathaniel Marchetti, Gerard J. Criner

**Affiliations:** 1 Departments of Thoracic Medicine and Surgery, the Lewis Katz School of Medicine at Temple University, 3401 North Broad Street, Philadelphia, PA 19140 USA; 2 Department of Pulmonary and Critical Care at St. Luke’s University Health Network, Philadelphia, PA 18015 USA; 3 Departments of Radiology, the Lewis Katz School of Medicine at Temple University, Philadelphia, PA 19140 USA; 4 Departments of Biostatistics, the Lewis Katz School of Medicine at Temple University, Philadelphia, PA 19140 USA

**Keywords:** Acute exacerbation, Combined pulmonary fibrosis and emphysema, IPF, COPD

## Abstract

**Rationale:**

Patients with combined pulmonary fibrosis and emphysema (CPFE) may develop acute exacerbations of IPF (AE-IPF) or COPD (AE-COPD). The incidence and the characteristics of exacerbations in patients with CPFE (e.g., COPD vs IPF) have not been well described.

**Objectives:**

To compare the incidence and rate of exacerbations in patients with CPFE vs. IPF and evaluate their effect on clinical outcomes.

**Methods:**

Comprehensive clinical data from CPFE and IPF patients were retrospectively reviewed. Baseline characteristics including lung function data, oxygen requirements, and pulmonary hemodynamics, were collected. Acute exacerbation events in both groups were defined clinically and radiographically. In the CPFE group, two patterns of exacerbations were identified. AE-COPD was defined clinically by symptoms of severe airflow obstruction causing respiratory failure and requiring hospitalization. Radiographic data were also defined based on previously published literature. AE-IPF was defined clinically as an acute hypoxic respiratory failure, requiring hospitalization and treatment with high dose corticosteroids. Radiographically, patients had to have a change in baseline imaging including presence of ground-glass opacities, interlobular septal thickening or new consolidations; that is not fully explained by other etiologies.

**Results:**

Eighty-five CPFE patients were retrospectively compared to 112 IPF patients. Of 112 patients with IPF; 45 had AE-IPF preceding lung transplant (40.18%) compared to 12 patients in the CPFE group (14.1%) (*p* < 0.05). 10 patients in the CPFE group experienced AE-COPD (11.7%). Patients with AE-IPF had higher mortality and more likely required mechanical ventilation and extracorporeal membrane oxygenation (ECMO) compared to patients with AE-COPD, whether their underlying disease was IPF or CPFE.

**Conclusions:**

CPFE patients may experience either AE-IPF or AE-COPD. Patients with CPFE and AE-COPD had better outcomes, requiring less intensive therapy compared to patients with AE-IPF regardless if underlying CPFE or IPF was present. These data suggest that the type of acute exacerbation, AE-COPD vs AE-IPF, has important implications for the treatment and prognosis of patients with CPFE.

## Background

The association between pulmonary fibrosis and emphysema was initially described in 1990 by Wiggins et al., who described eight heavy smokers with pulmonary fibrosis and upper lobe emphysema on High-resolution computed tomography scans (HRCT). These patients exhibited preserved lung volumes [1]. The term combined pulmonary fibrosis and emphysema (CPFE) was first used in 2005 by Cottin et al., who characterized a homogeneous group of patients with both emphysema and interstitial lung disease (ILD) with pulmonary fibrosis in the lower lobes [2].

However, since CPFE disease has been described, little is known about acute exacerbations of the disease, specifically, evaluating the incidence and the patterns of AE [3–5].

Thus far, there is conflicting evidence regarding the extent of emphysema and disease outcomes. A few studies have reported better survival rates in patients with IPF who have more extensive emphysema had than those who did not have emphysema, or those who had trivial emphysema [3, 6]. Several retrospective studies have contradicted this theory when they reported that the presence and extent of emphysema had no prognostic impact on survival of patients with IPF after correction for baseline disease severity [7, 8].

In fact, none of the studies have described the nature of the acute exacerbation events that occurs in patients with CPFE, and whether or not those affected patients’ outcomes. In one retrospective review published by Kurashima and his colleagues [3], he reported AE that led to death in 4/129 patients in the UIP/emphysema group compared to 20/233 patients in the UIP group. These were described as acute exacerbations of pulmonary fibrosis. In another single center cohort study in Japan whose aim was to evaluate predictors of AE in the CPFE group, 22/93 (24%) patients had AE during their observation period. This was defined similar to AE-IPF per the American Thoracic Society (ATS) criteria. All the patients who had AE died during their follow up period [4]. And finally, Inomata et al. examined the autopsy results of 22 patients with CPFE. They reported that 6 of these patients died of an acute exacerbation of interstitial pneumonia [5]. None of the above studies attribute acute exacerbations to the obstructive component of the CPFE disorder.

With the above information and given the coexistence of both emphysema and fibrosis in this single disease entity, we questioned whether AE of CPFE is attributed to the emphysematous or fibrotic component of the disease and what difference it would have on patient outcomes.

Herein, we compare the incidence and the rate of AE in CPFE vs IPF patients with end-stage disease. We also aim to define patterns of AE in the CPFE group, recognizing that they may experience either AE-COPD or AE-IPF and the clinical impact of AE on disease morbidity and mortality.

## Material and methods

### Study design and population

**Fig. 1:**
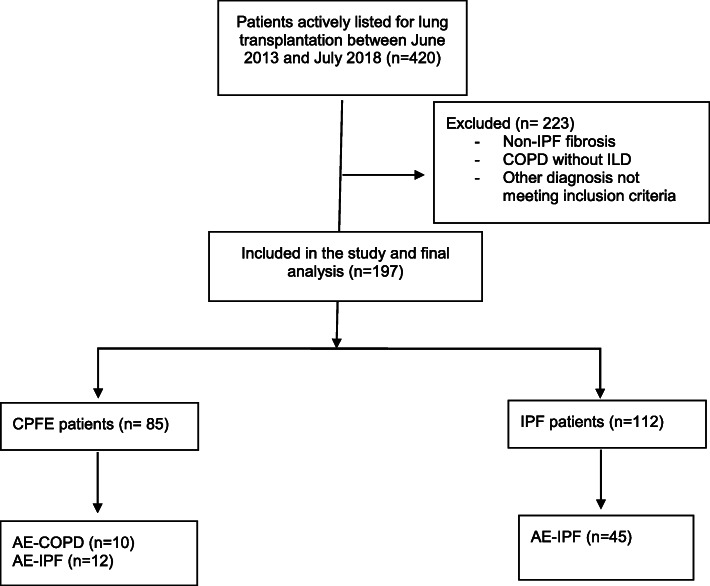
Study CONSORT diagram

This is a single center retrospective review that included all patients who met our inclusion criteria. The study was conducted at Temple University Hospital, which is one of the largest lung transplant centers in the US by the volume of surgeries performed each year. Between 120 to 145 lung transplant surgeries have been performed annually in the past 3 years for a variety of end stage lung diseases. In this review, we screened adult patients (age > = 18 years) who were listed for lung transplantation at Temple University Hospital between June 2013 and July 2018, and either received organ transplantation or died prior to that. Only patients with CPFE and IPF were selected for the study (Fig. 1).

### Definition of CPFE or IPF in the studied patient population

Patients with CPFE and IPF were identified for the review based on their clinical data and listing diagnosis given by their primary pulmonologists. Their diseases were then verified by the pathology (either from the explanted lungs or biopsy) and/or high-resolution computed tomography (HRCT) findings. For IPF patients, we followed the diagnostic criteria published by ATS/ERS guidelines [9, 10]. All patients had usual interstitial pneumonia (UIP) on their biopsy and/or HRCT findings consistent with UIP pattern without a clear etiology of their diseases. Currently, there is no consensus definition of CPFE syndrome, thus we considered the criteria published by Cottin et al. that was referenced in most of the CPFE literature; the presence of upper lobe emphysema and interstitial honeycombing on HRCT. On review of pathology of the explanted lung, these patients had both emphysema and UIP [2, 11]. We excluded patients with nonidiopathic interstitial pneumonias, history of connective tissue disease, histopathology other than UIP and patients with HRCT findings that are not consistent with UIP.

### Data collection and measures

All the data were collected using our electronic medical records from testing done during the year preceding lung transplant or death. This was done to assure that patients were in similar stage of their disease. The tests were done during their evaluation for lung transplantation. If multiple tests were done at our center, earliest results were taken. Baseline tests were performed when patients were clinically stable, and not during an acute exacerbation.

Demographics: These included age, gender, ethnicity, body mass index (BMI), presence or absence of smoking history and the number of pack years of tobacco use.

Pulmonary function testing: Spirometry, lung volumes and diffusion capacity of carbon monoxide was collected for each patient. All the tests were performed in the outpatient settings and were adequate for interpretation per ATS/ERS criteria [12, 13].

Oxygen use and blood gas data: Using the most recently performed six-minute walk test, we determined oxygen use for each of the patient at rest and on exertion and the six-minute walk test distance (6-MWTD) [14]. Baseline arterial blood gas data (PaCO_2_ and PaO_2_) were also collected from testing done on room air.

Pulmonary hemodynamics: Data regarding pulmonary hemodynamics were collected from reports of the right heart catheterization that was done as part of the transplant evaluation for each patient. These included mean pulmonary arterial pressure (mPAP), pulmonary capillary wedge pressure (PCWP), and pulmonary vascular resistance (PVR).

Cardiac function data, for patients in both groups, were reviewed. This was determined by the left ventricular ejection fraction (LVEF) reported on the baseline echocardiogram that was done during the year preceding lung transplantation.

### Acute exacerbations

We defined acute exacerbation events in each group clinically and radiographically. All the patients who were admitted for acute exacerbation in our study had a high-resolution chest CT (HRCT) as part of their work up. The HRCT images (baseline and during AE) were reviewed by a blinded experienced senior radiologist and an experienced pulmonologist.

In patients with underlying IPF, exacerbation events were identified (AE-IPF). In patients with underlying CPFE, acute respiratory events were determined to be either driven by emphysema and airflow obstruction (AE-COPD) or by fibrosis (AE-IPF).

AE-COPD in patients with CPFE was defined as per GOLD guidelines published in 2018, specifically, the severe category of AE; an acute worsening of respiratory symptoms that requires hospitalizations. Patients in this category are typically treated with bronchodilators and corticosteroids. This is manifested clinically by symptoms of airflow obstruction and acute bronchospasm [15]. We identified radiographic findings of AE-COPD that were adopted from the previously published literature. These included airway wall thickening, mucous impaction, atelectasis, consolidations and mediastinal adenopathy [16, 17].

In patients with underlying IPF or CPFE, AE-IPF was defined per the diagnostic criteria previously published by Collard et al. “an acute, clinically significant, respiratory deterioration characterized by evidence of new, widespread alveolar abnormality.” Clinically, these patients presented with an acute hypoxic respiratory failure, requiring hospitalization and treatment with high dose corticosteroids. Radiographically, patients had to have a change in baseline imaging including presence of ground-glass opacities, interlobular septal thickening or new consolidations on a background pattern consistent with a UIP; that is not fully explained by cardiac failure or fluid overload [18].

### Clinical outcomes

Our two co-primary outcomes were 1) the incidence and the rate of AE in CPFE patients compared to IPF and; 2) the patterns of AE in the CPFE group, i.e.; AE-IPF versus AE-COPD. The incidence of AE refers to the AE event that occurred during the follow-up period. The rate of AE for each group was calculated based on the number of AE events that met our definition criteria during the follow-up period (incidence rate of exacerbation per person-year). This was determined from the date patients were listed for transplantation to the date they received lung transplantation or date of death (whichever occurred first).

Our secondary outcomes included the impact of AE in both groups on disease morbidity and mortality. This was determined by the need for invasive mechanical ventilation, ECMO or both. Mortality was determined by death related to an acute exacerbation event during the follow up period.

### Data analysis

Data are presented as means SD for continuous variables or as percentages for categorical variables. We used chi-squared test for categorical data, and a two-sided t-test was used for continuous data. *P* values < 0.05 were considered statistically significant. The Fisher exact test was used for simple between-group comparisons. The software we used to run the statistics was Stata 14 (*Stata Statistical Software: Release 14*. College Station, TX: StataCorp LP).

Temple University Hospital Review Board approved the protocol.

## Results

### Baseline characteristics

85 CPFE patients were retrospectively compared to 112 IPF patients. All patients were listed for lung transplantation and met our inclusion criteria. Baseline characteristics are shown in Table 1.

**Table 1:** Baseline Characteristics

	CPFE (*n* = 85)	IPF (*n* = 112)	*P* value
Age (year)	66.4 (6.7)	66.7 (6.2)	0.72
Gender (F)	13 (15.29%)	31 (27.68%)	0.039
Ethnicity			0.225
Caucasian	75 (88%)	98 (87.5%)	
Hispanic	1 (1.2%)	6 (5.36%)	
African American	9 (10.6%	8 (7.14%)	
Prior History of Smoking	85 (100%)	66 (58.9%)	< 0.01
Pack-years	42.47 (20.2)	22.2 (11)	< 0.01
BMI (kg/m^2^)	29.4 (4.5)	27.9 (4.4)	0.02
FVC (L)	2.73 (0.79)	1.75 (0.55)	< 0.01
FVC %	69.2 (18.1)	45.6 (11.7)	< 0.01
FEV1 (L)	2.04 (0.6)	1.48 (0.46)	< 0.01
FEV1%	69.6 (22.5)	51.8 (13.8)	< 0.01
FEV1/FVC	74.0 (12.84)	84.54 (7.27)	< 0.01
TLC (L)	4.17 (1.2)	2.89 (0.7)	< 0.01
TLC %	66.1 (16.5)	47.17 (8.6)	< 0.01
RV (L)	1.4 (0.61)	0.93 (0.32)	< 0.01
RV (%)	59.24 (29.6)	41.63 (13.4)	< 0.01
RV/TLC	32.8 (7.3)	32.1 (9.0)	0.61
DLCO %	21.5 (8.2)	27.1 (11.5)	< 0.01
DLCO/VA %	39.34 (14.4)	57.14 (17.6)	< 0.01
PaO2 (mmHg)	55.9 (12.9)	54.78 (11.6)	0.53
PCO2 (mmHg)	37.23 (6.0)	39.81 (5.9)	0.04
O_2_ at rest (LPM)	3.14 (2.5)	3.23 (2.5)	0.79
O_2_ on exertion (LPM)	13.0 (3.6)	10.3 (3.9)	0.033
6-MWTD (meters)	260.2 (86.7)	260 (82)	0.98
LVEF (%)	61.5 (6.6)	62.2 (6.7)	0.47
Pulmonary HTN (%)	38 (44.7%)	33 (29.5%)	0.027
mPAP (mmHg)	28.43 (10.8)	23.5 (9.1)	< 0.01
PCWP (mmHg)	9.84 (5.5)	7.95 (4.8)	0.011
PVR (Wood units)	4.3 (3.2)	3.37 (2.1)	0.016

Patients with CPFE were all smokers with an average of 42.47(20.2) pack year history vs 22.2 (11.0) in the IPF group, *p* < 0.01. Patients with CPFE had significantly higher lung volumes and lower diffusion capacity of carbon monoxide (DLCO) compared to IPF patients; forced vital capacity (FVC) 2.73 (0.79) vs 1.75 (0.55) L, total lung capacity (TLC) 4.17(1.2) vs 2.89 (0.7) L, DLCO 21.5% (8.2) vs 27.1% (11.5), *p* < 0.01. More patients in the CPFE group had pulmonary hypertension compared to the IPF group, 38 (44.7%) vs 33 (29.5%), p 0.027 and their mean pulmonary arterial pressure group was significantly higher 28.43 (10.8) vs 23.5 (9.1) mmHg *p* < 0.01 (Table 1).

### Clinical outcomes

Of the 112 patients with IPF; 45 had AE preceding lung transplant (40.18%) compared to 22 patients in the CPFE group (25.8%), (p 0.023). The rate of acute exacerbations per person-year in both groups was similar; 0.284 in the CPFE group compared to 0.273 in the IPF group, *p* = 0.85.

10 (11.7%) patients in the CPFE group experienced AE-COPD and 12 (14.4%) had AE-IPF. The rate of AE-COPD was lower than patients with AE-IPF in the CPFE group (0.115 vs. 0.219, p 0.053).

Patients who experienced AE-IPF had significantly higher morbidity, requiring invasive mechanical ventilation and ECMO as compared to patients with AE-COPD, regardless if they had IPF or CPFE.

Mortality rate was not statistically different; 5/22 (22.7%) in the CPFE group and 14/45 (31.1%) in the IPF group, *p* = 0.47. All death events in the CPFE group were related to AE-IPF (Table 2).

**Table 2:** Exacerbation Data and Clinical Outcomes in Patients with CPFE and IPF

	CPFE (*n* = 85)		IPF (*n* = 112)	*P* value
Acute Exacerbation preceding lung transplantation	22/85 (25.8%)		45/112 (40.18%)	0.023
	AE-COPD	AE-IPF		* < 0.01
	10/85 (11.7%)	12/85 (14.1%)		
Rate of exacerbation per person-year	0.284		0.273	0.85
	0.115	0.219		*0.13
				**0.053
Need for invasive mechanical ventilation	10/22 (45%)		20/45 (44.44%)	0.8
	2/22 (9%)	8/22 (36%)		* < 0.01
Need for invasive mechanical ventilation and ECMO	3/22 (13.6%)		17/45 (37.8%)	0.043
	0/22 (0%)	3/22 (13.6%)		* < 0.01
Exacerbation-related mortality	5/22 (22.7%)		14/45 (31.1%)	0.47

* *P* value comparing AE-IPF in the CPFE group vs AE-IPF in the IPF group

** *P* value comparing AE-IPF and AE-COPD in the CPFE group

Patients with AE-IPF in the CPFE and IPF groups had similar radiographic findings including new GGOs (85%), interlobular septal thickening (100%) and/or new consolidations (15%). Patients with CPFE and AE-COPD were most likely to have airway wall thickening (60%), adenopathy (50%) and/or new areas of patchy consolidations (30%).

## Discussion

In retrospective review of our patient population, patients with CPFE had a significant smoking history, preserved lung volumes and more pulmonary hypertension compared to patients with IPF similar to what prior studies have reported [2–4, 19, 20]. In our primary outcome, we found that CPFE patients were less likely to experience an acute exacerbation event prior to lung transplantation compared to IPF patients. This might be partially related to the fact that emphysema contributes to preservation of lung volumes, which perhaps makes these patients less prone to having alveolar collapse and recurrent atelectatic lung injury, a well-established hypothesis in the pathogenesis of IPF disease [21].

An interesting remark of our study was that the incidence rate of acute exacerbations per person-year in both groups was not significantly different. Similarly, even though the rate of AE-IPF in the CPFE group was less compared to the one in the IPF group, this was not statistically significant. However, the rate of AE-COPD was significantly less compared to AE-IPF in the CPFE group.

**Fig. 2:**
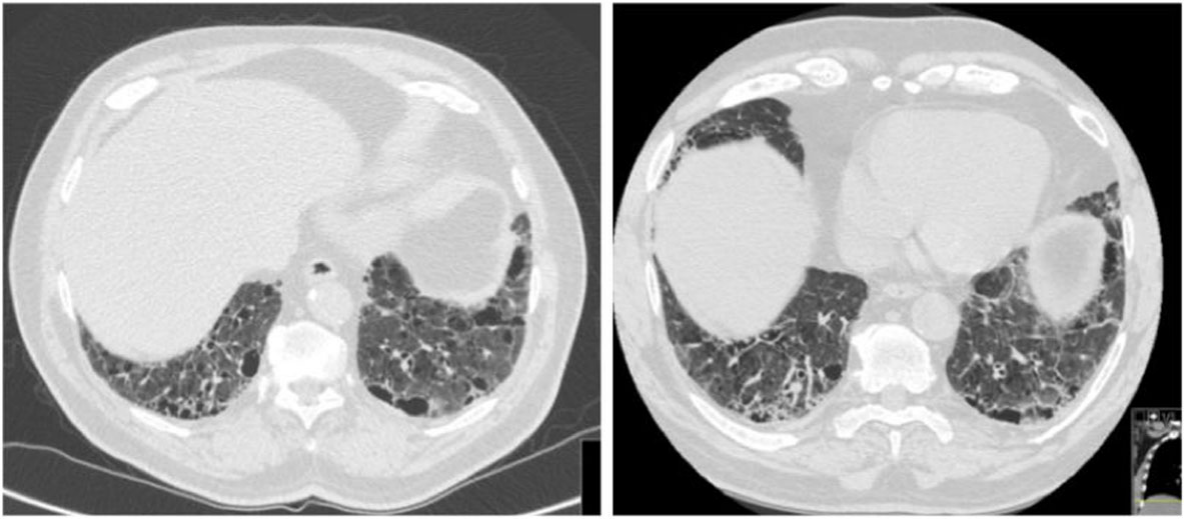
HRCT of CPFE patient. Baseline (left) and during AE-COPD (right). Findings include peribronchial wall thickening; note absence of GGOs

**Fig. 3:**
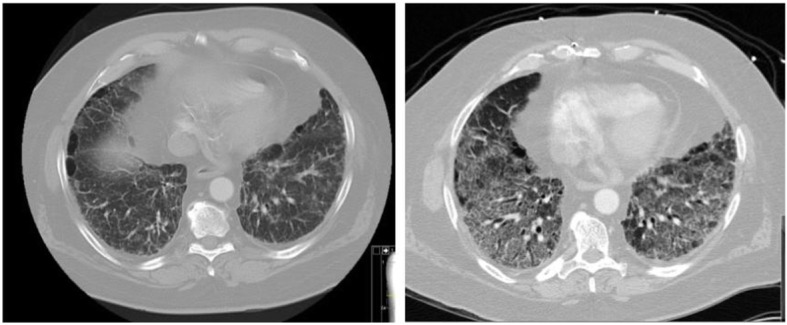
HRCT of CPFE patient. Baseline (left) and during AE-IPF (right). Findings include GGOs and interlobular septal thickening

In our study, we aimed to make a distinction between CPFE patients who experienced AE-COPD and the ones who had AE-IPF. This distinction is based on clinical and radiographic findings that are different between the two groups (Table 3, Figs. 2 and 3). This is important as the treatment and the clinical consequences are different in these two groups. Patients with CPFE and AE-COPD had better outcomes, requiring less invasive therapy, including invasive mechanical ventilation and/or ECMO compared to CPFE patients with AE-IPF.

**Table 3:** Radiographic and Clinical Parameters used to Define Acute Exacerbations in patients with CPFE

Characteristics	Acute Exacerbations in CPFE	
	AE-COPD	AE-IPF
Baseline Imaging (HRCT)	Fibrosis and Emphysema are equal in predominance	Fibrosis predominance Fibrosis and Emphysema are equal in predominance
Clinical Findings	Acute worsening of respiratory symptoms (airflow obstruction, increased sputum production and acute bronchospasm)	Acute hypoxic respiratory failure that is not explained by cardiac dysfunction, infection or fluid overload.
Radiographic Findings	Airway wall thickening Lymphadenopathy Consolidations	New GGO Interlobular septal thickening Consolidations
Treatments	Bronchodilators Corticosteroids +/− Antibiotics	High dose corticosteroids +/− Antibiotics
Outcomes	Mechanical ventilation	Mechanical ventilation +/− ECMO

In addition, we found a strong correlation between baseline pattern on HRCT findings and the pattern of AE. After reviewing baseline imaging for the CPFE patients, we attempted to visually classify a predominant pattern; emphysema, fibrosis or neither (equal predominance). We noticed that patients with emphysema predominant pattern were the least likely to experience AE. On the other hand, patients who tend to have equal predominance of both emphysema and fibrosis may experience either AE-COPD or AE-IPF. The majority of patients who experienced AE-IPF had predominant fibrosis on their baseline imaging. It is important to mention that this was a clinical impression made by both the radiologist and the pulmonologist. We think that this could be an interesting future area of investigation.

This above observation, along with the characteristic radiographic findings that we have defined, might suggest that HRCT may be a helpful tool to evaluate patients during the AE event in patients with CPFE as it may provide treatment guidance, and prognostic information in these patients.

Interestingly, the above data posits the question whether CPFE is really a separate entity or is the result of two different lung diseases that are simultaneously active in patients with a significant history of prior smoking. The radiographic pattern and the clinical outcomes most likely will depend on which disease process is more predominant and/or more active at the present time [22, 23].

The main limitation of our study is due to its retrospective, single center observational design. We were limited in capturing hospitalizations outside our health systems which might underestimate the rates of AE in each group. In addition, patients were only captured after being listed for lung transplantation. Thus, timing for referral could have affected the study findings as any AE prior to patient’s referral was not captured. Although we understand that our results may not be generalizable for mild or moderate disease stages, however, IPF clinical course remains unpredictable, and AE can occur in patients with preserved lung function [24].

However, our study has some important strengths. We studied a higher volume of subjects compared to other studies that have reported on clinical data in patients with CFPE [2, 5, 8, 20]. We included a homogenous group of consecutive patients that are in a similar stage of their diseases (all were listed for lung transplantation at our institution). In addition, we were able to identify clinical and radiographic phenotypes of the types of acute exacerbations that may occur in patients with CPFE.

To our knowledge, this is the first study that makes a distinction between acute exacerbations of COPD or IPF and looks specifically at the clinical impact of acute exacerbation events in the CPFE patients.

## Conclusion

In summary, our study suggests that CPFE patients may experience either AE-IPF or AE-COPD. Patients with CPFE and AE-COPD had better outcomes, requiring less intensive therapy compared to patients with AE-IPF, whether the underlying disease was CPFE or IPF. This indicates that the type of acute exacerbation in CPFE patients, has important implications for the treatment and prognosis of the disease. These data provide meaningful information to clinicians that care for patients with CPFE.

## Data Availability

The datasets used and/or analyzed during the current study are available from the corresponding author on reasonable request.

## Abbreviations

IPF: Idiopathic Pulmonary Fibrosis
COPD: Chronic obstructive pulmonary disease
AE: Acute exacerbation
CPFE: Combined pulmonary fibrosis and emphysema
UIP: Usual interstitial pneumonia
GOLD: Global Initiative for Chronic Obstructive Lung Disease
HRCT: High-resolution Computed tomography
BMI: Body-mass index
ATS: American Thoracic society
ERS: European Respiratory Society
PFT: Pulmonary Function Testing
FEV1: Forced Expiratory Reserved Volume in the first second
FVC: Forced Vital Capacity
RV: residual Volume
TLC: Total Lung Capacity
DLCO: Diffusion Capacity of Carbon Monoxide
DLCO/VA: Diffusion Capacity of Carbon Monoxide divided by Alveolar Volume
6-MWTD: Six-minute walk-test distance
PVR: Pulmonary vascular resistance
mPAP: Mean pulmonary arterial pressure
PCWP: Pulmonary capillary wedge pressure
PaO2: Partial pressure of oxygen
PaCO2: Partial pressure of carbon dioxide
LVEF: Left ventricular ejection fraction
ECMO: Extra-corporeal membranous oxygenation
GGO’s: Groundglass opacities
